# To Pair or not to Pair? Machine-Learned Explicitly-Correlated Electronic Structure for NaCl in Water

**DOI:** 10.1021/acs.jpclett.4c01030

**Published:** 2024-05-31

**Authors:** Niamh O’Neill, Benjamin X. Shi, Kara Fong, Angelos Michaelides, Christoph Schran

**Affiliations:** † Yusuf Hamied Department of Chemistry, University of Cambridge, Lensfield Road, Cambridge CB2 1EW, United Kingdom; ‡ Cavendish Laboratory, Department of Physics, University of Cambridge, Cambridge CB3 0HE, United Kingdom; § Lennard-Jones Centre, University of Cambridge, Trinity Ln, Cambridge CB2 1TN, United Kingdom

## Abstract

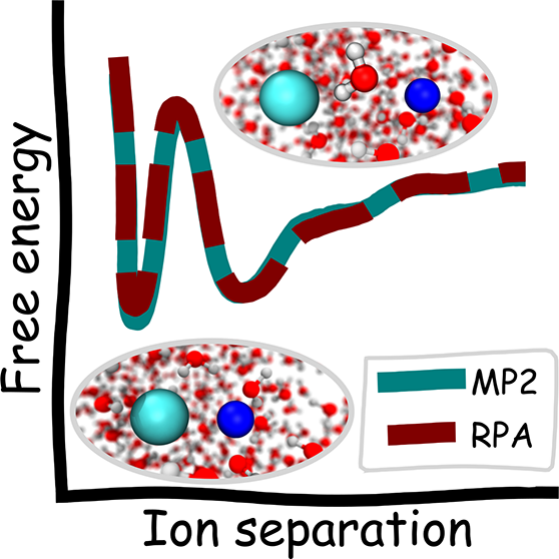

The extent of ion pairing in solution is an important phenomenon to rationalize transport and thermodynamic properties of electrolytes. A fundamental measure of this pairing is the potential of mean force (PMF) between solvated ions. The relative stabilities of the paired and solvent shared states in the PMF and the barrier between them are highly sensitive to the underlying potential energy surface. However, direct application of accurate electronic structure methods is challenging, since long simulations are required. We develop wave function based machine learning potentials with the random phase approximation (RPA) and second order Møller–Plesset (MP2) perturbation theory for the prototypical system of Na and Cl ions in water. We show both methods in agreement, predicting the paired and solvent shared states to have similar energies (within 0.2 kcal/mol). We also provide the same benchmarks for different DFT functionals as well as insight into the PMF based on simple analyses of the interactions in the system.

Understanding the nature of ion pairing and the solvation structure of electrolyte solutions is a fundamental challenge in the quest to design efficient next generation energy storage devices.^1^ For example, ionic conductivity and redox stability are predominantly influenced by the electrolyte solvation structure.^2,3^ Moreover, an understanding of the solvation behavior of ions is crucial to ensure a uniform and stable solid–liquid interphase to limit dendrite formation, which currently presents a significant challenge with respect to efficiency and safety of electrochemical devices.^4^ More generally for transport properties, Peng et al. showed that the diffusion of sodium ions at interfaces is significantly affected by their hydration number.^5^

The potential of mean force (PMF) between an ion pair in solution provides a direct window into the ion pairing behavior and solution structure of electrolytes. However, for the prototypical electrolyte solution of NaCl in water, no experimental benchmark exists. Moreover, from a computational modeling perspective, it is highly sensitive to the underlying potential energy surface^6,7^ and there is currently no quantitative or qualitative consensus on the PMF of NaCl in water. For example, two of the best-established classical force fields for NaCl in water—the Joung-Cheatham (JC)^8^ and Smith-Dang (SD)^9^ models—both disagree significantly in their PMFs, with the JC model predicting thermodynamically unfavorable ion pairing.^10,11^ In general, electrolytes are very challenging to model, where on top of the already arduous situation for bulk water,^12^ they have the additional complication of ion–water and ion–ion interactions that need to be accurately described.^13^ Therefore, it is clear that, to rationalize the PMF, it is imperative to first have a model that accurately describes the structure of NaCl in water.

The past decades have seen pioneering work in the development of classical force field models to describe ions in water, with the most recent Madrid scaled charge models for ions giving excellent agreement with experimental data for a range of structural and dynamical properties.^14^ However, in general, such models can typically lack accuracy beyond the property or phase space to which they have been parametrized. It has been suggested that to simultaneously and faithfully capture dynamical and structural properties of electrolytes, a model that explicitly treats its electronic structure is desirable.^15,16,16^

Until now, the workhorse method of *ab initio* computational chemistry—density functional theory (DFT)—has been the method of choice for studying these systems, offering an acceptable balance of accuracy and computational overhead. While the solvation structure of water around ions has been the subject of numerous previous *ab initio* simulation studies,^17−22^ there is currently no consensus on a sufficiently accurate model to describe NaCl in water. In fact, both Duignan et al. and Panagiotopoulos and Yue have recently highlighted the urgent need for accurate *ab initio* models for electrolytes to capture their dynamics^16^ and collective properties.^6^

For example, tried and tested exchange-correlation (XC) functionals for liquid water, such as the generalized gradient approximation (GGA) revPBE-D3, are not guaranteed to perform well when ions are added.^19,20,23^ This is because standard GGAs tend to perform poorly for interactions involving charges, overestimating electrostatic contributions to binding energies^24^ along with their well-known delocalization error,^25,26^ both arising from the self-interaction error. Moreover, the interplay of the electronic structure method and nuclear quantum effects has been shown to be highly sensitive for liquid water,^27−29^ but their impact on electrolyte solutions has not been exhaustively explored so far.^30,31^ Inclusion of more complex ingredients into the density functional approximation following Perdew’s Jacob’s Ladder^32^ may improve the description of water–water^33,34^ or ion–water^35^ interactions; however, a model that faithfully captures the behavior of ions in solution for the right reasons remains elusive.

Going beyond DFT, correlated wave function-based methods such as the random phase approximation (RPA) and second-order Møller–Plesset perturbation theory (MP2) are expected to perform well for electrolyte systems. These methods naturally incorporate van der Waals interactions and do not suffer from delocalization error.^36^ They have shown initial promise for liquid water, accurately predicting the correct relative densities for ice and water, which is governed sensitively by a balance of van der Waals and hydrogen bonding interactions.^37−39^ While Duignan et al. hint that RPA could outperform the lower rungs of Jacob’s Ladder for the case of the solvated sodium ion,^20^ routine application of these high-level methods in condensed phase simulations has been sporadic. This is primarily because of their high computational cost to implement, with canonical scaling behavior between  and , albeit reduced-scaling variants also exist.^40^ Meanwhile, to obtain statistically converged properties to probe the structure of electrolyte solutions including radial distribution functions, densities, and solvation free energies, even DFT becomes extremely computationally challenging. While valuable developments in electronic structure code algorithms and computer hardware^38,41^ have significantly increased the accessibility of these methods, it would be highly desirable to confine them to a small number of single-point energy (and force) calculations rather than finite temperature simulations, which typically require hundreds of thousands of such computations.

Fortunately machine learning potentials provide a gateway to perform simulations at *ab initio* levels of theory with a decrease in several orders of magnitude in their computational cost.^42,43^ Machine learning models have been successfully trained and applied to study bulk water at various levels of theory,^44−48^ including recent neural network models for bulk water with MP2^49,50^ and RPA.^51^ While the natural next step is to add ions to the water,^52−54^ this brings additional complexity to the configuration space to be explored, and so the training set must be judiciously chosen to reflect this. To this end, we use a previously developed automated active learning framework^55^ and an initial training set describing NaCl dissolution in water^56^ to generate MLPs at various levels of electronic structure theory for Na and Cl ions in water.

The forthcoming discussion will simply refer to all of these models by the name of the reference method to which they have been trained. We believe this is valid since we have rigorously benchmarked the capability of the MLPs to reproduce their underlying reference method (see Supporting Information (SI)). We first explore the capabilities of different DFT XC functionals and correlated wave function methods to accurately describe the structure of ions in water, showing that both RPA and MP2 generally reproduce experimental densities and radial distribution functions for both bulk water and solvated ions. We then use these wave function based methods as a benchmark against which to compare the predictions of DFT and classical force fields for the potential of mean force of a NaCl ion pair in water. We provide simple metrics based on the constituent interactions to rationalize the overall shape of the PMF, while also providing insight into the ability of DFT to capture these interactions. In all cases, the simulations performed are well-converged with respect to statistical sampling and are not significantly impacted by finite-size effects, highlighting a major advantage of the MLP approach over *ab initio* methods. We finish with an outlook on the potential of these models for describing more complex situations such as confined electrolytes and for computing dynamical properties.

In Figure 1, we compare with experimental measurements the performance of various DFT XC functionals, along with two wave function based methods MP2 and RPA, in predicting the radial distribution functions (RDFs) of both bulk water and Na and Cl ions in water. The DFT functionals have been chosen to span the various levels of Jacob’s Ladder, with each level incorporating increased complexity into the XC functional description. Specifically we have chosen the GGA revPBE-D3,^60,61^ the van der Waals inclusive optB88-vdW,^62^ the meta-GGA r^2^SCAN,^63^ and the hybrid revPBE0-D3.^61^ Beyond DFT, we consider RPA and MP2, with both classical and quantum nuclei. Inclusion of nuclear quantum effects have been shown to be necessary to obtain accurate agreement with the experiment for liquid water structural and dynamical properties, including RDFs, diffusion coefficients, and spectroscopy.^27,33^

**Figure 1:**
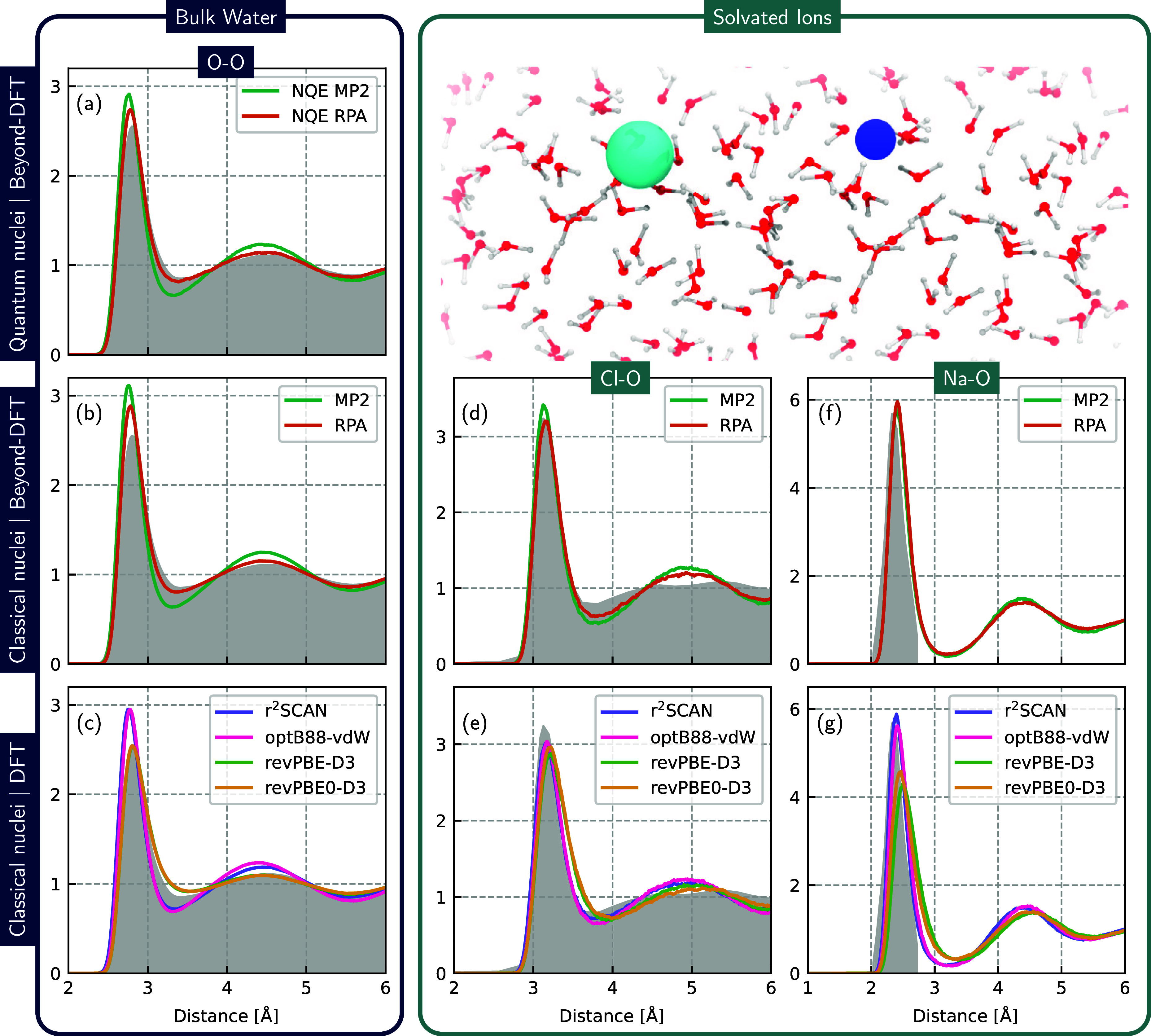
Comparison of radial distribution functions with experiment for correlated wave function methods and various DFT XC functionals. The first column shows the O–O RDF for bulk water, considering nuclear quantum effects with RPA and MP2 (a), classical nuclei for RPA and MP2 (b), and classical nuclei for DFT (c). Columns 2 and 3 show the O–Cl and O–Na RDFs, respectively, of a solvated NaCl ion pair in water for RPA and MP2 with classical nuclei (d and f) and DFT with classical nuclei (e and g). Experimental references in each case are shaded in gray. The bulk water O–O experimental RDF is taken from ref (57). Cl–O is neutron scattering data from ref. (58). for KCl. The Na–O reference from ref. (19) is the rescaled peak from X-ray diffraction data.

We first consider the performance of the RPA and MP2 wave function-based methods. Overall, out of all methods tested, RPA performs best in describing the structure of both bulk water and Cl and Na ions in water (Figure 1a, d, and f, respectively), accurately reproducing experimental RDFs. For bulk water, the first peak height of the O–O RDF with RPA and classical nuclei (b) is overestimated by approximately 16%. Inclusion of nuclear quantum effects (a) reduces the height of the first peak compared to classical nuclei (b), yielding excellent agreement with the experiment, with just a slight overstructuring of the first peak, consistent with previous literature.^51^ Meanwhile, MP2, even with quantum nuclei, shows some deficiencies for bulk water, predicting a more structured liquid by overestimating the height of the first peak by approximately 11%, underestimating the first minimum also by approximately 11% and also predicting greater long-range order than the experiment. The poorer performance of MP2 for bulk water is consistent with previous literature. Lan et al. showed that MP2 predicts overstructured water and a lower diffusion coefficient compared to the experiment,^49^ attributing some of the shortcomings in that study to an incomplete basis set, while Willow et al. in ref (64) also show that MP2 predicts denser water than experiment and DFT under ambient conditions.

Upon comparing correlated wave function simulations with classical and quantum nuclei (see the SI), it seems that the ion–water structure is more forgiving with respect to NQEs than bulk water for both RPA and MP2, with no significant improvement to the structural description when NQEs are included. Both RPA and MP2 with classical nuclei are in excellent agreement with experiment for the Na–O and Cl–O RDFs, accurately reproducing the position and height of the first peak. Simulations that include NQEs result in essentially identical RDFs for these cases, as shown in the SI. It should be noted that only the first peak of the Na–O RDF was quoted from experimental X-ray diffraction (XRD) measurements.^19^ Also, the experimental Cl–O RDF is only available from a KCl solution.^58^ While there are discrepancies beyond the first peak, the cation should not significantly influence the first solvation peak in the large separation limit.

The challenges of accurately modeling bulk water *and* ions in solution for DFT is apparent when comparing the different DFT XC functional predictions for the RDFs with those of the experiment. None of the functionals tested can simultaneously describe water and ions with the same accuracy as the best performing correlated wave function method, RPA. As has been previously observed, revPBE-D3 performs very well for liquid water,^27,65^ however it has been shown that this is in part due to a fortuitous cancellation of errors and this breaks down upon inclusion of NQEs.^27^ Moreover, while it also performs well for the Cl–O RDF, it significantly underestimates the first peak of the Na–O RDF. Inclusion of a fraction of exact exchange via the hybrid revPBE0-D3 functional shows similar good performance for bulk water and Cl–O but does not improve the performance for Na–O. In contrast, r^2^SCAN significantly overstructures bulk water, predicting a first RDF peak for O–O approximately 20% greater than the experiment, yet it shows good agreement with the experiment for both ion types in water. Various density corrections to the r^2^SCAN functional have shown to improve its description of liquid water,^66^ which warrant further tests on their suitability for aqueous electrolytes. Similar to r^2^SCAN, the van der Waals inclusive optB88-vdW functional also overstructures bulk water but accurately predicts the ion–water RDF for both Na–O and Cl–O. The poor performance for both revPBE-D3 and revPBE0-D3 for Na–O can be potentially ascribed to the fact that the D3 correction does not account for changes in dispersion due to charge-transfer effects (i.e., the formation of ions). Cations have been shown to have a significantly different polarizability (i.e., dispersion) than their neutral atom counterpart compared to anions,^67^ thus explaining the greater impact on sodium. More sophisticated treatment of these cases such as the D4 correction,^68^ methods incorporating iterative Hirschfeld partitioning,^69^ and using van der Waals inclusive methods (as shown here with optB88-vdW) may alleviate this problem. It has also been suggested that simply neglecting the D3 correction for the problematic cation interactions can improve agreement with experiment.^70^

Accurately predicting the density is another important benchmark of the ML models and their underlying electronic structure reference method. Figure 2a compares the concentration-dependent density prediction of RPA and MP2 with experiment. This is a stern test of the electronic structure method but also the MLP quality, covering a significant concentration range. Both MP2 and RPA show a similar qualitative increase in density with concentration of NaCl as observed in the experiment. MP2 is within 1% of the experimental values, while RPA more significantly overestimates the density by approximately 5%. Both RPA and MP2 MLP predictions for the bulk water density are also in close agreement with previous *ab initio* predictions of 0.994 g/cm^3^^37^ and 1.020 g/cm^3^^39^ for RPA and MP2, respectively, and are within 5% of the experimental value. Figure 2b compares the DFT and the wave function MLP predictions of the density of a 2 M NaCl solution with the experimental value. There is again a wide spread in DFT predictions, with most functionals tested overestimating the density of the 2 M NaCl solution apart from revPBE0-D3, which underestimates the density by 5% compared to the experiment, while r^2^SCAN is in good agreement with experiment. In comparison, RPA slightly overestimates the density, while MP2 is in very good agreement with experiment. It should also be noted that while classical force-field models can accurately reproduce experimental density predictions, particularly in low concentration regimes,^71^ its agreement arises because the density is a property to which the water model has been explicitly fitted.^72^ At higher salt concentrations, away from the regions explicitly used in the parametrization, force-field predictions also deviate from the experiment.^71^ To summarize, although RPA gives excellent structural properties for NaCl in water, it is slightly outperformed by MP2 in terms of the density response with increasing NaCl concentration. However, MP2 yields poorer agreement with the experiment for structural properties than RPA. Nevertheless, the overall commendable all-round performance of RPA and MP2 for all cases of ions and water suggests them as reliable methods with which to study NaCl in water. Of course the enhanced computational cost for initial training set computations for both RPA and MP2 over DFT should be mentioned, however this is a one-off investment during training. Once a model is obtained, it can then be applied in simulations with a reduction of several orders of magnitude in computational cost—where the savings are greater for models based on more expensive methods.

**Figure 2:**
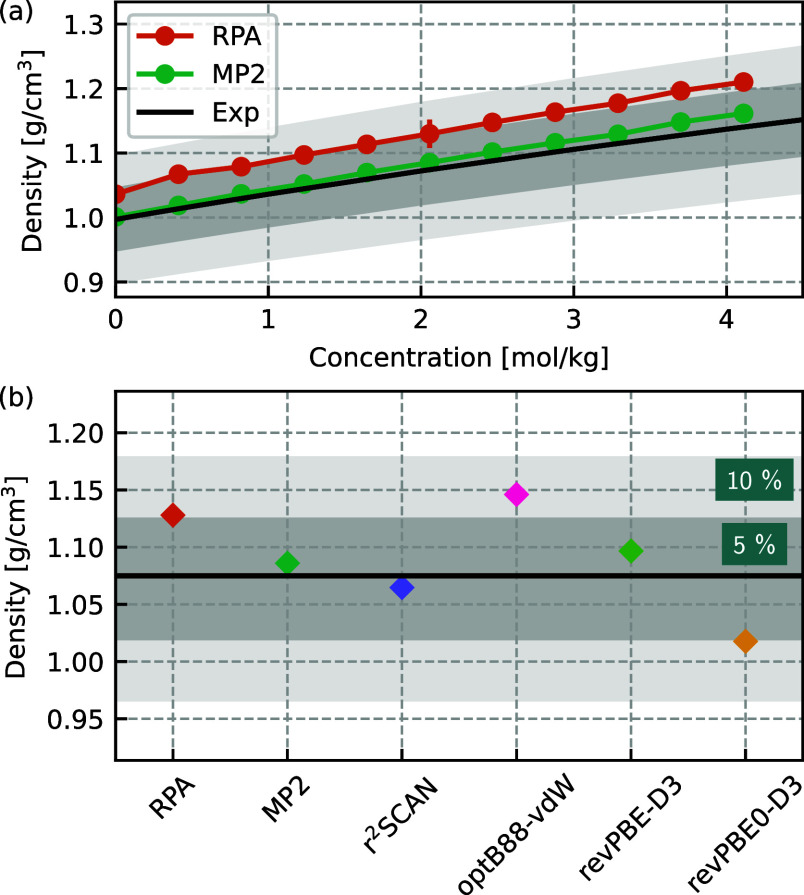
Comparison of densities with the experiment^59^ for different DFT XC functionals and beyond DFT methods. Panel a shows the NaCl concentration-dependent density for RPA and MP2 at 300 K. Panel b compares the experimental prediction at 300 K for a 2 M NaCl solution. In both cases, a 5 and 10% boundary around experiment is shown shaded in dark and light gray, respectively, to facilitate comparisons.

Going beyond experimentally accessible properties, the potential of mean force (PMF), denoted here as *w*(*r*), is a fundamental property of the electrolyte governing the extent of ion pairing in solution and capturing collective solvent motion. The key features of this quantity for Na^+^ and Cl^–^ in water are two local minima corresponding to the so-called contact–ion pair (CIP) and solvent-shared ion pair (SSIP), with free energies *U*_CIP_ and *U*_SSIP_, respectively. These are separated by a barrier (TS) along the interionic separation coordinate as illustrated in Figure 3a, where *U*_b_ is defined as *U*_TS_ – *U*_CIP_. The relative stabilities of these minima (Δ*U*_SSIP–CIP_ = *U*_SSIP_ – *U*_CIP_), along with the barrier height (*U*_b_), are crucial to understanding the kinetics of ion-pair association and dissociation.^74^ However, as previously mentioned, the PMF is highly sensitive to its underlying potential energy surface.^6,7^ Among both DFT and classical models, there is a major unresolved question regarding the relative stabilities of the CIP and SSIP,^10,73,75,76^ with some classical models predicting a more stable CIP by up to 4 kcal/mol compared to DFT, which generally predicts almost degenerate CIP and SSIP states. It should also be noted that the error bars on the DFT predictions are typically much greater, due to the significant computational effort required to converge the PMF. Despite commendable efforts to resolve the PMF using *ab initio* methods,^7,73,75,77^ it is computationally intensive to statistically converge, requiring long simulation times (∼2–3 ns) and several replicates to obtain statistical error bars. Therefore, in the absence of experimental benchmarks, a reference PMF based on a high-level electronic structure method is particularly valuable to the community. It can provide clear atomistic insight into explaining experimental observations of ion pairing and allow comparison among theoretical models—both *ab initio* and classical.

**Figure 3:**
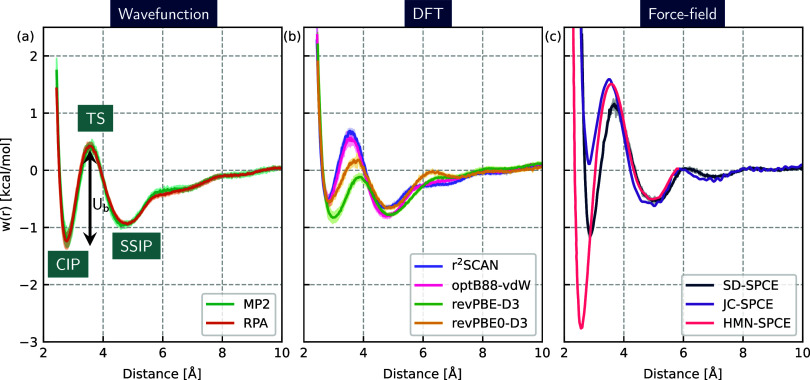
Potential of mean force for the Na Cl ion pair in water with associated statistical error bars for various MLPs and classical force fields: (a) wave function methods RPA and MP2, with contact ion pair (CIP), transition state (TS), solvent-shared ion pair (SSIP), and barrier height (*U*_b_) annotated. (b) DFT and (c) classical force fields. HMN-SPCE is taken from ref (73) and JC-SPCE from ref (10).

The MLPs developed in this work are ideal for efficient and statistically converged calculation of the NaCl PMF at a given level of theory. We compute the PMF via the Na–Cl RDF of a 1 M NaCl solution comprising six ion pairs in a box of 332 waters. This highlights another advantage of the MLP approach, where the potential energy surface along the interionic separation can be sufficiently sampled through standard molecular dynamics simulations, without the requirement for enhanced sampling methods such as thermodynamic integration (we show in the SI the equivalence of both methods). Moreover, these models have enabled sampling of the PMF out to the large-separation limit, providing valuable insights into the ion-pair binding energies.

Overall, over 200 ns of MD simulations were performed, which is significantly beyond the capabilities of *ab initio* methods. Figure 3 compares the PMF predictions for MLPs at the same levels of electronic structure theory described above, as well as some commonly used classical force fields. Statistical convergence and finite size effects have been carefully investigated, and details are given in the SI. We first note that the correlated wave function methods MP2 and RPA are in very good agreement, predicting that the CIP and SSIP have very similar stabilities. They both predict that the two states are within ∼0.2 kcal/mol of each other. This resolves the so far unanswered question regarding the relative stabilities of the CIP and SSIP states. Moreover, of all the electronic structure methods tested, MP2 and RPA predict the largest barrier between the CIP and SSIP states of roughly 1.6 kcal/mol. The excellent agreement between both high-level methods suggests this PMF can now be used as a benchmark to compare other methods.

Similar to the RDFs and densities shown above, DFT predictions also vary significantly (although not to the same extent as classical force fields) and reinforce the considerations for selecting a suitable functional to study electrolyte systems. While all methods are in relatively good agreement (within 0.2 kcal/mol) for the relative stabilities of the CIP and SSIP states, there is a larger range in the predictions of the barrier from SSIP and CIP and the depth of the CIP state. The free energy barrier between the CIP and SSIP for both r^2^SCAN and optB88-vdW is similar at approximately 1.25 kcal/mol. Meanwhile, although revPBE-D3 and revPBE0-D3 predict similar barrier heights for the CIP to SSIP transition, revPBE-D3 shows a shift to slightly larger distances for the locations of the CIP, SSIP, and transition state. Given the wide-ranging performance of the DFT methods tested here with respect to various aspects of the electrolyte structure (shown in Figures 1 and 2), it is perhaps unsurprising that they also struggle to concur on the PMF, a property that depends sensitively on the collective interactions of sodium and chlorine with the surrounding water.

We now rationalize these trends in the PMFs by a heuristic analysis of the subtle balance of interactions that qualitatively match our findings. We focus on the two key interactions, namely, the ion–ion and ion–water interactions. We compute the binding energy of an ion pair in a vacuum up to 3 Å (before the transition to SSIP), to understand ion–ion (II) effects. For ion–water (IW) effects, we compute the energy of interaction for an ion pair in water from equilibrium periodic snapshots taken from the points of interest along the PMF (CIP, SSIP, TS). Details of these calculations as well as further comparisons are given in the SI. Figure 4 shows the relationships between these constituent interactions and the PMF observables. First, in Figure 4a, there is a strong correlation between the depth of the CIP minimum in the PMF and the difference of the ion–water and ion–ion interactions. We show in the SI that considering the ion–ion interactions alone is not the best descriptor for the CIP well depth. However, we find that subtracting the ion–water interactions from the ion–ion interactions as shown here effectively captures the trend of CIP well depth across the functionals. All functionals except optB88-vdW show the same ordering for both properties (within error bars), suggesting the CIP region is primarily governed by a delicate interplay between ion–ion and ion–water interactions, whereby stronger ion–ion (ion–water) interactions strengthen (weaken) the CIP well depth. However, the deviation of optB88-vdW from this trend suggests that there still may be important yet subtle effects for example from water–water interactions in the close contact region. This analysis builds on work by Duignan et al.^20,78^ who compute interaction energies from clusters extracted from simulations of cations in water. In Figure 4b, there is again a clear correlation between the barrier going from the CIP to SSIP states and the strength of the ion(-pair)–water interaction energy. Here, within error bars, the largest transition barrier corresponds to the largest ion–water interaction energy. We rationalize this observation by the fact that going from the SSIP to CIP state requires a rearrangement of the ions’ solvation shell. A lower ion–water interaction energy will thus result in a more facile transition from one minimum to the other, thereby lowering the barrier between the two states.

**Figure 4:**
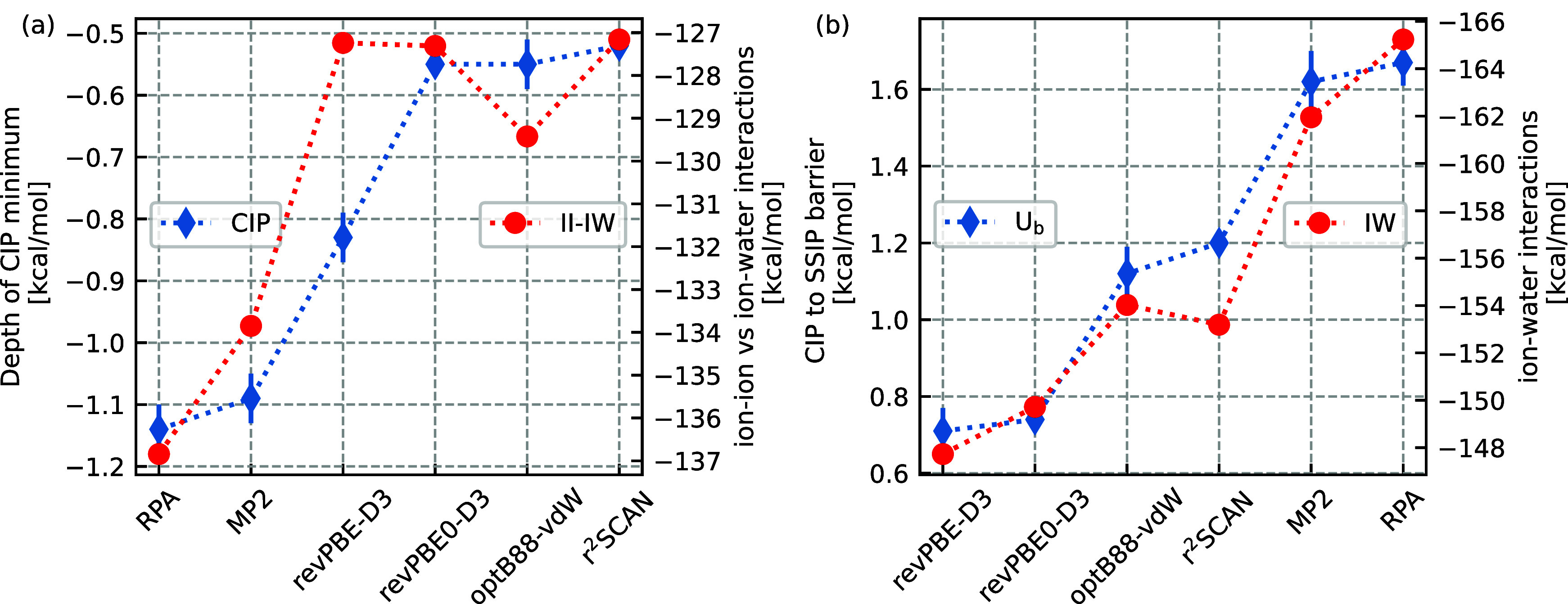
Relationship between constituent interactions in the Na^+^–Cl^–^ solution and PMF observables. (a) The depth of the CIP minimum (CIP) is strongly correlated to the balance of the ion–ion vs ion–water interactions, whereby we have subtracted the ion–water interactions from ion–ion interactions (II-IW). (b) The barrier height between the CIP and SSIP (*U*_b_) is strongly correlated with the strength of the ion–water interactions (I–W).

These qualitative metrics can also be further used to understand the performance of the various electronic structure methods employed here. First, for the ion–ion interactions, MP2 and RPA both show the strongest binding compared to DFT.^79,80^ With respect to the ion–water interactions, MP2 and RPA also exhibit the strongest interactions, with the accuracy of the former having been verified by previous hydrated sodium cluster analysis.^81^ Meanwhile, revPBE-D3 and revPBE0-D3 demonstrate the weakest ion–water interactions. We show in the SI that while the Na^+^–H_2_O interaction is overbound due to the D3 (as discussed previously), there is an underbinding in the Cl^–^–H_2_O interaction. This arises from the revPBE exchange enhancement factor causing stronger repulsion, tending to underbind,^82^ which then dominates the overall ion–water interaction. In agreement with analyses on hydrated sodium ion clusters by Duignan et al.,^20^ we find that r^2^SCAN (or SCAN in their work) gives better agreement to RPA than revPBE-D3.

Finally, the largest discrepancies in PMFs from all the classes of methods tested are in the classical force-field predictions. The Smith-Dang/SPCE model^9^ is similar to the MP2 and RPA prediction, with the position of the SSIP slightly shifted to larger distances and a larger barrier by roughly 0.4 kcal/mol. However, the Joung-Cheatham/SPC/E and HMN/SPC/E models significantly differ from all the other tested methods. JC predicts a more stable SSIP by almost 1 kcal/mol, while HMN/SPCE predicts a more stable CIP by over 2 kcal/mol, and a barrier of over 4 kcal/mol between CIP and SSIP. As shown in the analysis of interactions above, given the sensitive dependence of the PMF on the interplay of interactions, this perhaps explains why classical force fields, which are typically fitted to observables rather than the interactions may struggle to converge on the PMF.

The machine learning approach used in this work offers a high level of accuracy and efficiency for studying electrolyte solutions, yielding high-quality, well-converged structural properties. We compute the disputed potential of mean force of the NaCl ion pair in water using correlated wave function methods. We find that correlated wave function methods are in very good agreement for the description of structural properties and densities. Importantly, they also agree that the CIP and SSIP are essentially degenerate (within 0.2 kcal/mol). DFT can reproduce these results with larger variations depending on the chosen functional, where we do not identify a functional that delivers convincing performance throughout the chosen properties. Compared to the scale of force field predictions, in particular for ion-pairing, these differences remain small and candidates such as revPBE-D3 or r^2^SCAN are expected to deliver a good compromise when simulating extended system sizes. Going forward, the remaining differences among DFT XC functionals and even the discrepancies that remain among the wave function based methods point toward a need for an unambiguous benchmark quality model for these systems. Given the recent successes by Chen et al.^83^ and Daru et al.^84^ in performing CCSD(T)-based—considered the “gold-standard” of quantum chemistry—molecular dynamics for bulk water, there is an exciting opportunity to build on this work for the case of ions in water. Their machine learning models accurately predict structural and dynamical properties of liquid water compared to the experiment. Using these approaches to train a model for water including ions is therefore a promising prospect to obtain a high quality potential energy surface for this system that can be used to further benchmark the lower level methods and also to be directly used for simulations.

Furthermore, we have explored the effect of the various interactions of the system on the PMF, providing insight into the delicate interplay of ion–ion and ion–water interactions. The simple metrics can now be used to benchmark other electronic structure or force field methods, using for example future CCSD(T)-based models as references. From a more general perspective, our work highlights how ML potentials can now be used to efficiently screen various levels of theory directly on properties of interest as has also been recently shown for the case of perovskite phase transitions.^85^

Beyond simply comparing levels of theory, the PMFs and models generated in this work can next be used to provide valuable insights into the experiment and computationally measured dynamical properties of electrolytes. Obtaining accurate dynamical properties remains a frontier of research in the field of electrolyte simulations. There has been significant work using *ab initio* data to parametrize continuum scale models of electrolytes to access quantities such as osmotic coefficients and activities, along with transport properties such as diffusion coefficients and conductivity.^6^ This work shows that using MLPs offers an alternative yet complementary approach in the quest to obtain a fully *ab initio* description of electrolytes. In Figure S.10 of the Supporting Information, we report the water self-diffusion coefficients as a function of concentration relative to pure water for RPA and MP2 in good agreement with experiment, in particular for MP2. We note that at the accurate electronic structure level employed in this work other factors such as the quantum nature of the nuclei^29^ start to become a dominating factor to reach quantitative agreement with the experiment, as shown recently for pure water.^27,45^ The here developed machine learning potentials combined with path integral molecular dynamics enable addressing this issue in future studies via efficient sampling of the potential energy surface. From a fundamental perspective, building on the work of Geissler et al., a high level reference model is imperative to establish a quantitative understanding of the kinetics of ion pair dissociation.^74^ Similarly, a reliable model capable of accurately describing the bulk structure of electrolytes is primed to target dynamical properties of interest, including activity and osmotic coefficients, along with transport properties such as diffusion coefficients and conductivity. Relating the PMF to Onsager transport coefficients, which quantify correlations in ion motion,^86,87^ would be highly insightful to rationalize various transport phenomena, and an interesting first step would be to compute standard ion pair association energies.^88^ In particular, the ionic conductivity as a measure of the quantity of current an electrolyte can transport is vital for the development of next generation energy storage devices.^89^ Another important consideration and open question for these problems is the influence of long-range effects, given the large simulation cells required to compute these properties and the long-range electrostatic interactions between ions. More sophisticated long-range schemes than that used in this work to name but a few include refs (90−92), and a comparison of their performance would be highly insightful. Moreover, recent equivarient graph-based architectures including MACE,^93^ NEQUIP,^94^ and GRACE^95^ are by construction longer range and are again highly promising to address the question of long-range interactions in an efficient manner. Beyond the bulk, confinement of electrolytes leads to interesting physics and unexpected phenomena that are highly relevant to a range of applications, including blue energy harvesting^96^ and desalination.^97,98^ In particular, extension of our current models to explore the intriguing phenomenon of confinement-induced ion pairing is very attractive. Recent work has shown the assembly of confined ions in solution into long chains under an electric field, resulting in the so-called “memristor” effect,^99^ for which accurate atomistic insights are urgently needed.^100^

## Methods

### Machine Learning Potential

MLPs provide a direct mapping between a structure and its energy and forces, bypassing the computationally costly step of solving the Schrödinger equation for each time step of a simulation. All of the models in this work are based on the seminal work of Behler and Parrinello,^101^ where we train a committee of eight neural network models^47^ for Na and Cl ions in water on forces and energies computed at various levels of electronic structure theory. The model is systematically trained over three generations. The first comprises a general training set common to all models obtained from previous work^56^ for NaCl ions in water. We then use an active learning procedure^55^ screening different solution concentrations to augment the training set for each model to ensure relevant configurations for a given level of theory are included to give the second generation model used in production simulations. The third generation ensures that the large ion–ion separation limit is accessible, by performing another active learning scheme on nonisotropic simulations cells or transfer learning depending on the specifc model. To address the question of long-range forces in our models, the final model is trained only on short-range energies and forces, where the long-range electrostatic term (based on fixed point charges assigned to each atom and computed by particle mesh Ewald summation) has been subtracted. This Coulombic baseline is then added on during simulations to give the full forces and energies. Complete technical details of the models’ training and validation are given in the SI.

### Molecular Dynamics Simulations

All classical simulations were carried out using the CP2K/Quickstep code at a constant temperature of 300 K maintained using the Canonical Sampling Through Velocity Rescaling (CSVR) thermostat^102^ and with a 1 fs time step.

### PMF

The PMF calculations were carried out with a 1.0 mol/kg NaCl solution comprising six NaCl ion pairs in 332 waters. This was first equilibrated in the NpT ensemble to obtain the equillibrium density for a given level of theory. To ensure sufficient statistical sampling, between five and 10 uncorrelated configurations were then sampled from this trajectory and used as independent initial conditions. Production simulations were then performed for at least 2 ns in the NVT ensemble at the previously computed equilibrium density, after which the radial distribution functions were obtained. The potential of mean force *w*(*r*) between Na and Cl was computed using the relation *w*(*r*) = −*k*_B_*T* ln *g*(*r*_Na–Cl_), where *k*_B_ and *T* are the Boltzmann constant and temperature, with *g*_Na–Cl_(*r*) being the radial distribution function between Na and Cl ion pairs. The final result was given by the average of these runs, with the standard deviation providing an error estimate. We show in the SI that this approach is fully consistent with performing constrained molecular dynamics with thermodynamic integration and does not suffer from finite size effects.

### RDFs

The RDF calculations for bulk water were obtained from simulations of 126 water molecules in the NVT ensemble at the experimental density. RDFs for ions were obtained from simulations of a single ion pair and 95 water molecules. Path integral simulations for RPA and MP2 were performed using ring polymer molecular dynamics with 16 replicas, using the PILE thermostat.^103^

### Electronic Structure

Electronic structure calculations were all carried out using the CP2K/Quickstep code. A plane-wave cutoff of 1200 Ry was required to converge the PMF (see the SI for detailed convergence tests), and a TZ quality basis set was used for each model. Further details of specific electronic structure settings for various levels of theory are given in the SI, including convergence tests for the various electronic structure settings.

## Data Availability

All data required to reproduce the findings of this study are available at GitHub (https://github.com/niamhon/nacl-water). All simulations were performed with publicly available simulation software (n2p2, CP2K), while the active learning package is available at GitHub (https://github.com/MarsalekGroup/aml-dev).

## Supporting Information Available

Electronic structure settings and convergence tests, details on machine learning potential development, simulation details and additional results including diffusion coefficients, interaction analysis, and rdfs (PDF)
