# Supplementary material for: To Pair or not to Pair? Machine-Learned Explicitly-Correlated Electronic Structure for NaCl in Water
Source: J Phys Chem Lett. 2024 May 31;15(23):6081–91. doi: 10.1021/acs.jpclett.4c01030 (PMC11181334; doi:10.1021/acs.jpclett.4c01030)
Supplement: Supplementary file 1 — jz4c01030_si_001.pdf [file jz4c01030_si_001.pdf]

Supporting Information for: To Pair or not to Pair? Machine-Learned Explicitly-Correlated Electronic Structure for NaCl in Water

Niamh O'Neill,[†,‡,¶] Benjamin X. Shi,[†,¶] Kara Fong,[†,¶] Angelos Michaelides,[*,†,¶] and Christoph Schran[*,‡,¶]

†*Yusuf Hamied Department of Chemistry, University of Cambridge, Lensfield Road, Cambridge, CB2 1EW, UK*

‡*Cavendish Laboratory, Department of Physics, University of Cambridge, Cambridge, CB3 0HE, UK*

¶*Lennard-Jones Centre, University of Cambridge, Trinity Ln, Cambridge, CB2 1TN, UK*

E-mail: am452@cam.ac.uk; cs2121@cam.ac.uk

Electronic Structure

DFT settings

All DFT calculations were performed in CP2K to generate both total energies and nuclear gradients.[S1] The electronic density was partitioned into core and valence contributions, with core electrons described using the norm-conserving Goedecker, Teter and Hutter (GTH) pseudopotentials.[S2] Na 2s and 2p electrons were also treated explicitly given the well-known issue of non-linear core-valence exchange/correlation. Valence electrons were described using the MOLOPT TZV2P basis set.[S3] To cover the range of DFT XC functional approximations, functionals were chosen from ascending rungs of Jacob's Ladder. In short we used the generalised gradient approximation, revPBE,[S4,S5] meta GGA r^{2}SCAN[S6] which improves on the numerical instabilities of SCAN,[S7] van der Waals inclusive optB88-vdW[S8] and hybrid revPBE0.[S9] revPBE and revPBE0 were used with with the zero-damping variant of Grimme's D3 dispersion correction.[S10] The revPBE0 calculations were performed using the auxiliary density matrix method,[S11] to reduce the cost of computing the exact exchange component.

Figure S1 shows the convergence of the forces on the four atom types with respect to plane-wave cutoff. A large cutoff is required to converge the forces on Na. Using the Gaussian and Augmented Plane Wave (GAPW) method[S12] resolves this issue, with forces converging after 400 Ry. However, the GAPW method is not available for wavefunction methods RPA and MP2. We therefore consider the effect of these stochastic forces on the sodium atoms with respect to the property of interest in this work - the potential of mean force of Na and Cl in water. Figure S2 shows the PMF for 3 machine learning potential (MLP) models at revPBE-D3 level of theory using the GPW method with increasing plane-wave cutoff. This property is well-converged with PW cutoff, with an acceptable error of approximately 0.1 kcal/mol. Additionally, Figure S3 shows that again the target property, the PMF is not affected by using the GPW method over the GAPW method. Therefore in summary, all DFT calculations were performed using the GPW method with 1200 Ry plane-wave cutoff.

Correlated wave-function theory

Random-phase approximation (RPA) and second-order Møller Plesset perturbation theory (MP2) were performed in CP2K[S1] to generate both total energies and nuclear gradients. We used DFT with the PBE functional as the starting point for the RPA correlation energy calculations. The resolution-of-identity (RI) techniques was used for these methods.[S13] We used triple-zeta (TZ) quality correlation consistent basis sets for H and O (taken from CP2K's `RI_cc_TZ` set) and correlation consistent basis sets designed by Ye and Berkelbach[S14] for Na and Cl. Auxiliary basis sets for the RI integral operations were generated using the automatic auxiliary basis of Stoychev et al.[S15,S16] for Na and Cl, with the defaults (from CP2K) used for H and O. A planewave cutoff of 1200 Ry was again used. We used the GTH-HF pseudopotentials from Goedecker-Teter-Hutter[S2] for all of the atoms. Figure S4 shows the force convergence on the atoms with respect to the GPW integral cutoff. A cutoff of 300 Ry was shown to be well converged within 0.00001 meV/Å. The calculation is also sensitive to the number of quadrature points and so 20 were used, which has an error of $<$ 1 mHartree according to literature.[S17]

Development and validation of machine learning potential

Automated work flow

The procedure for developing the committee neural network potentials (C-NNP) used in this work was followed as described in Ref S18. Overall 6 models were trained to describe NaCl ions in water at different levels of electronic structure theory (revPBE-D3, optB88-vdW, r^2SCAN, revPBE0-D3, MP2 and RPA). The training of the individual models was divided into three generations, and is graphically depicted in Figure S5. The first generation comprised a common training set for all models, adapted from previous work.[S19] Specifically, only

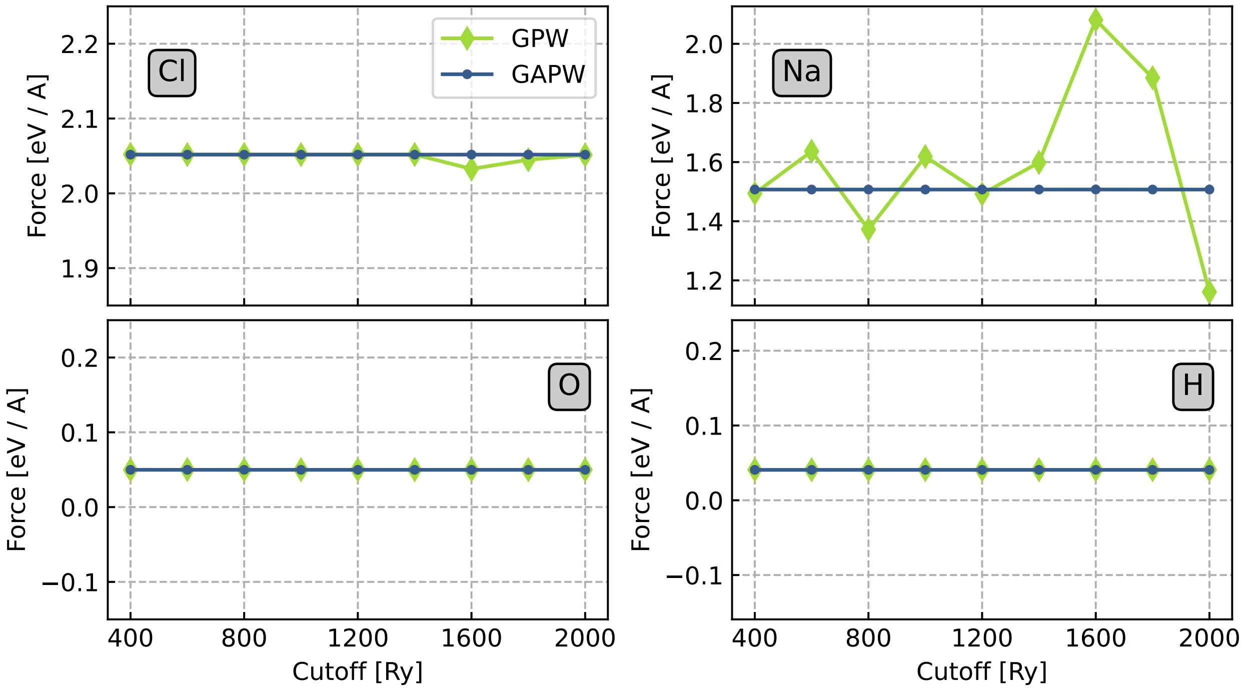

Figure S1: Convergence plots of forces on each atom type (Na, Cl, O, H) vs plane wave cutoff for GAPW (blue) and GPW (green) methods.

configurations corresponding to ions in solution were used, since this previous model also contained solid NaCl in water, which was used to explore dissolution. These configurations comprised increasing concentrations of NaCl ions in water (See Figure S5 for specifics). The forces and energies of this common training set were then computed for the different levels of theory (See Section for the electronic structure details) used in this paper, and individual models then trained as described below. In order to ensure the relevant configuration space for a given level of theory was suitably covered by the models, NpT simulations were then performed with each model for all of the solution concentrations used in Generation 1. An active learning procedure[S18] was then employed to select relevant configurations from each concentration to add to the model training set. For a given active learning iteration, 20 random structures from a reference trajectory were used to initialise the model. After training 8 NNP members, forces and energies of 2000 randomly selected structures from the reference trajectory were predicted to ascertain the force and energy committee disagreements. 20 structures with the largest mean force disagreement were added to the training set for

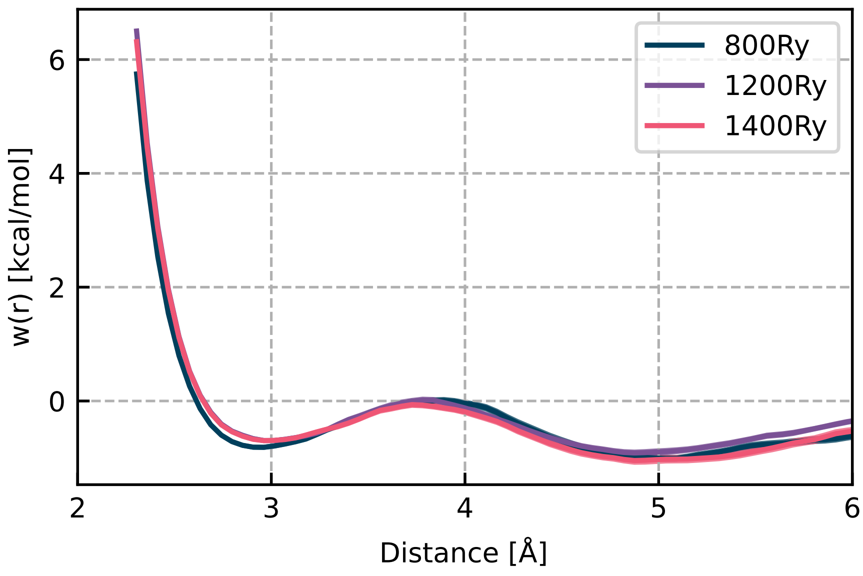

Figure S2: PMF for revPBE-D3 MLP with PW cutoffs at 800, 1200 and 1400 Ry.

the next round of active learning. Convergence was reached when new structures added to the training set did not improve the committee disagreement between points already in the training set, indicating the training set was sufficiently diverse. Overall, approximately 200 additional structures were added per model. In order to ensure the models could treat large ion-ion separations (beyond the 6 Å cutoff of the model), a third iteration of training was performed (Generation 3 in Figure S5). This generation entailed two different protocols depending on the electronic structure method. For revPBE0-D3, r^{2}SCAN and optB88-vdW, additional NpT simulations were performed on non-isotropic simulation cells of initial dimensions 10 x 10 x 20 Å containing 1, 2 and 3 ion pairs in 64, 62 and 60 waters respectively. Another active learning procedure as described above was performed, to add approximately 150 structures in total per model, leading to the training set of each model comprising $\sim$ 1100 structures (See Figure S5) for model-specific details. For RPA, MP2 and revPBE-D3, for reasons of computational cost (RPA and MP2) and computational complexity (revPBE-D3), a transfer learning protocol was employed to ensure that the second generation models could treat the large ion-ion separations. Transfer learning is a well-established machine learning technique, and has been successfully used in recent work for example in training a CCSD(T)

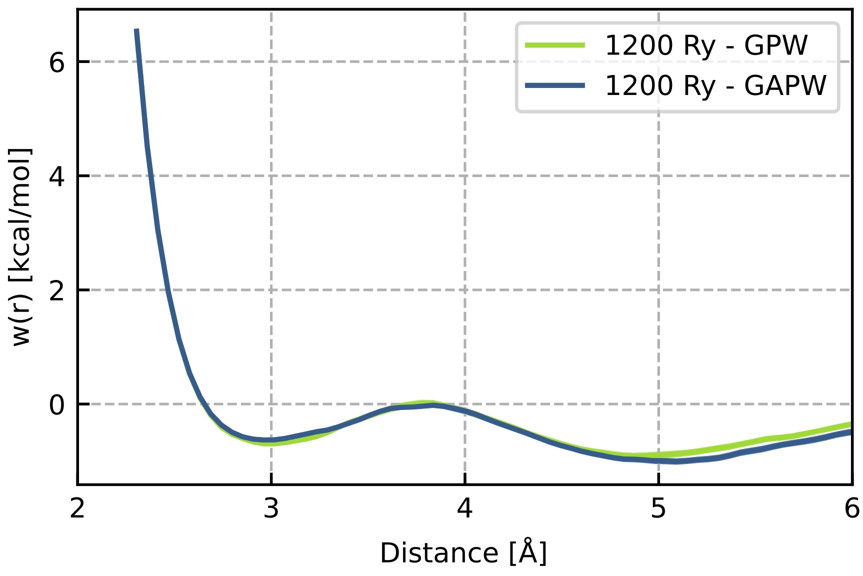

Figure S3: PMF for revPBE-D3 MLP with 1200 Ry PW cutoff for both GPW and GAPW methods.

water model with sparse training data.[S20] In this approach, the third generation r^2SCAN model was used as a baseline for the training of the RPA, MP2 and revPBE-D3 models. In this way, the training was initialised from the pre-optimised r^2SCAN weights (rather than randomly seeded weights as is conventionally done) using the relevant training set from the second generation. The specific benefits of this approach for our work are two-fold, typically requiring less training data and also easing the fitting procedure.[S21]

Details of Model

The chemical environment around each atom was described using a general set of atom-centered symmetry functions.[S22] There are 10 radial and 4 angular functions for each pair and triple of atoms, following Ref. S18. All symmetry functions used a cutoff function of angular cosine form with a cutoff radius of 12 Bohr. The committee was comprised of 8 NNP members, of identical architecture with 2 hidden layers and 25 neurons in each layer. In all cases, random sub-sampling was performed to introduce variability between the committee members, where 10% of the total set of structures were discarded. The weights and biases of

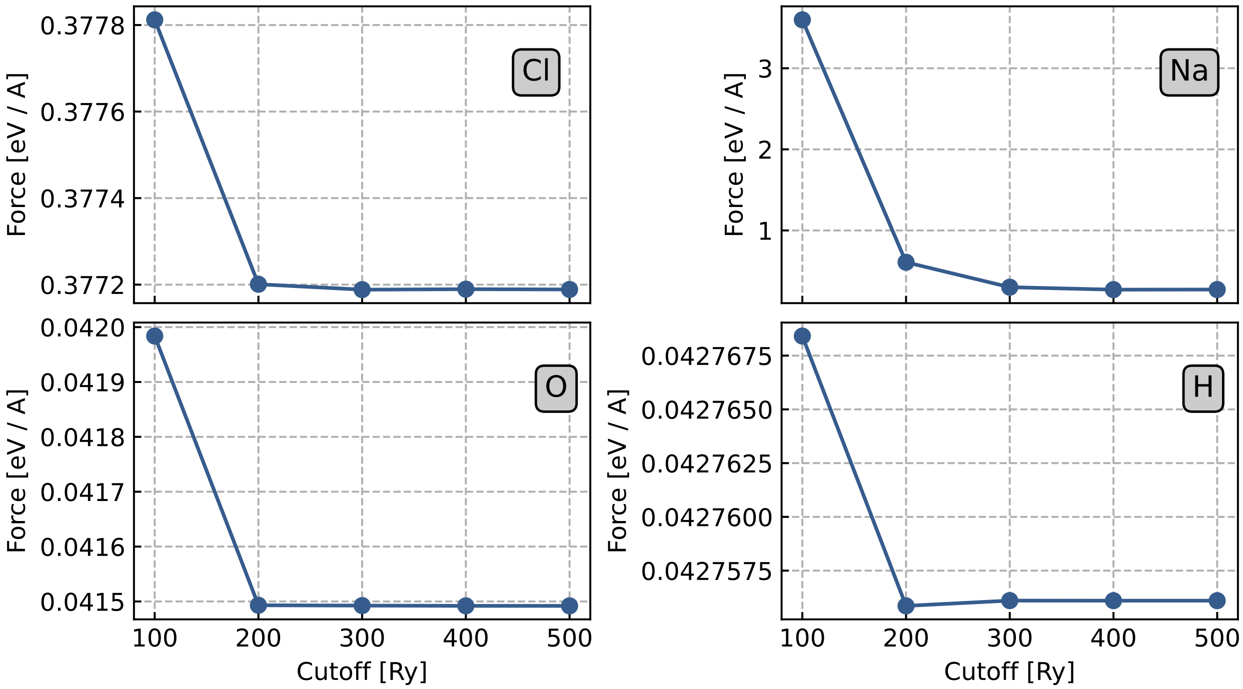

Figure S4: Convergance plots of forces on each atom type (Na, Cl, O, H) vs integral plane wave cutoff.

the NNPs were optimised using the n2p2 code.[S23] Individual models during active learning were optimised for 15 epochs, while the final C-NNP model used in simulations was optimised for 50 epochs.

We have explicitly incorporated long-range effects beyond the cutoff of the symmetry functions (12 Bohr) of the machine learning potential. The predicted energy can in general be written as a sum of short range and long range contributions (E_{sr} and E_{coul} respectively): $E_{\text{tot}} = E_{\text{sr}} + E_{\text{coul}}$. The long-range model was thus trained on the difference between the standard short-ranged model and the Coulomb contribution, calculated using point charges of +/- 1 respectively for Na and Cl and using TIP3P model parameters for water.[S24] We used this model in all production simulations, where the Coulomb contributions were explicitly included via particle mesh ewald summation. Details on the validation of the final models are presented in the next Section.

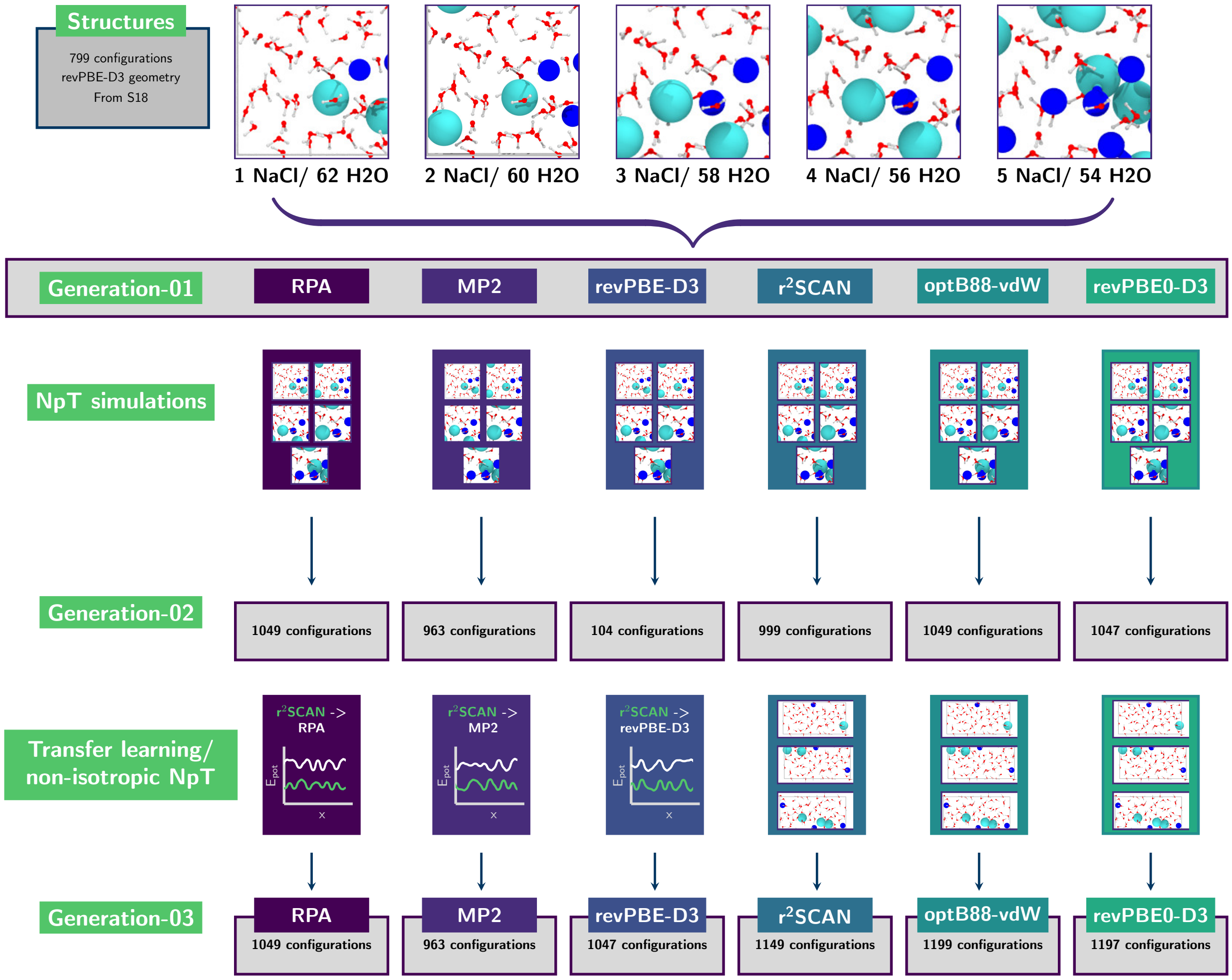

Figure S5: Schematic of training procedure for the 6 ML models in this work.

Validation

We validate the ability of the model to reproduce its underlying reference method based on a validation set of 100 configurations. These comprise a scan along the inter-ion separation coordinate up to 6 Å (giving a computationally reasonable box size for RPA and MP2 reference calculations). The force and energy RMSE for each model are shown in Table S1 for each model, and a representative correlation plot for r^{2}SCAN in Figure S6. These errors compare favorably with our previous model[S19] with RMSE values for both forces (37.0 meV/Å) and energies (0.3 meV/atom) as well as similar reactive systems, which have been studied using machine learning potentials, such as the work by Behler et al. in Ref. S25

who quote a force and energy RMSE for a model describing proton transport at ZnO/H_2O interfaces of 140.4 meV/Å and 1.0 meV/atom respectively.

Training errors, forces

Table S1: Summary of force and energy RMSE for each ML model.

Model	Energy RMSE [meV/atom]	Force RMSE [meV/Å]
revPBE-D3	2.366	27.280
optB88-vdW	0.503	37.435
r^2SCAN	0.407	41.100
revPBE0-D3	0.780	39.430
MP2	0.763	40.160
RPA	0.819	38.327

Simulation Details

System setup

The NaCl PMF was obtained from molecular dynamics simulation using the machine learning potentials from above. The system comprised 6 NaCl ion pairs in 332 waters giving a concentration of 1 mol/kg in a cubic simulation cell with periodic boundary conditions in the x, y and z directions. For each model, a 1 ns NpT simulation was first performed to obtain the equillibrium density. From the NpT simulations, 10 configurations were sampled to give uncorrelated starting configurations for production simulations run at the model density. Production simulations were performed in the NVT ensemble, with a timestep of 1 fs at 300 K. The PMF was then obtained from the average of the rdfs from the 10 independant simulations, using the relation $w(\mathbf{r}) = -k_B T \ln g(\mathbf{r}_{\mathrm{Na-Cl}})$ where k_B and T are the Boltzman constant and temperature (300 K), with $g_{\mathrm{Na-Cl}}(r)$ being the radial distribution function between Na and Cl ion pairs. The standard deviation between replicates was used to quantify the statistical uncertainty. Overall, over 200 ns of *ab initio* quality simulations were performed, highlighting the major advantage of the machine learning approach. The

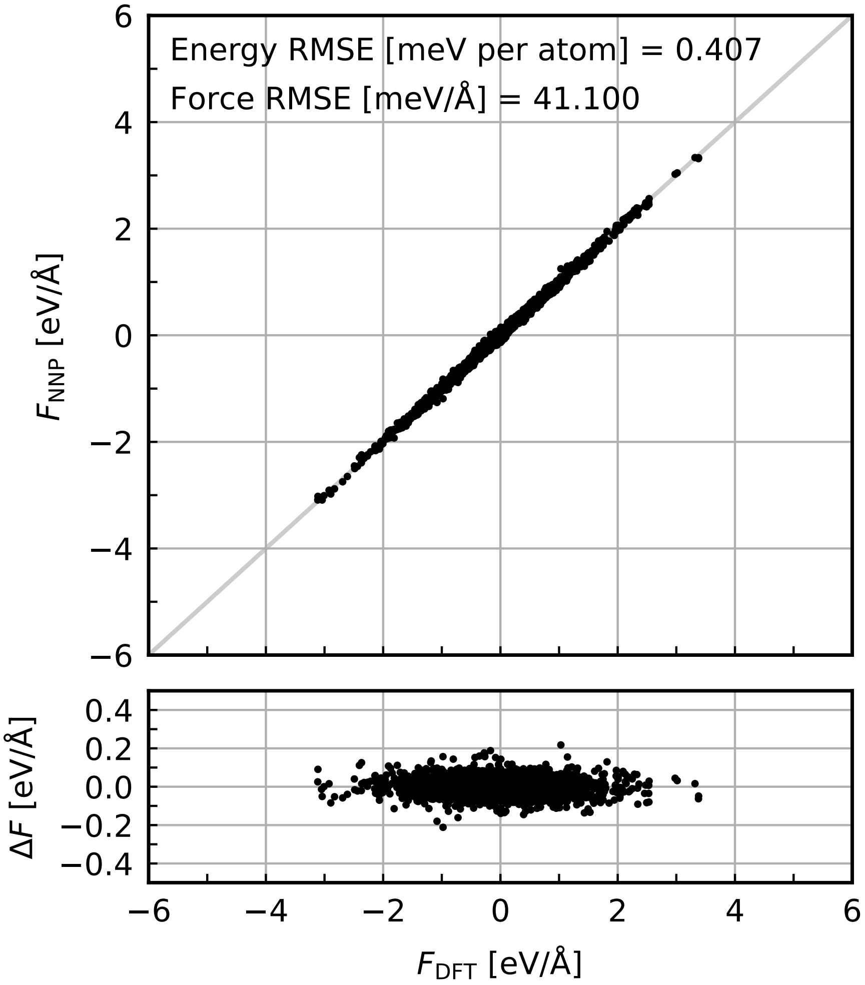

Figure S6: Correlation plot for r^{2}SCAN C-NNP predicted forces and corresponding reference DFT forces, with light grey line showing a perfect correlation coefficient of 1.

density and diffusion coefficients were computed as a function of concentration for MP2 and RPA. For a fixed number of 270 waters, increasing numbers of ion pairs were added to the box up to a final concentration of roughly 4 mol/kg (corresponding to 20 ion pairs in 270 waters.) The density was computed from an NpT simulation at each concentration, with a block averaging procedure to obtain statistical error bars. The diffusion coefficients were computed from NVT simulations at the equillibrium density obtained from the previous step, and production simulations were at least 4 ns long. Diffusion coefficients (D) were computed from the Einstein relation

$$D = \lim_{t \to \infty} \frac{1}{6} \frac{d \langle |\mathbf{r}(t) - \mathbf{r}(0)|^2 \rangle}{dt} \tag{S1}$$

The standard Yuh and Hummer correction for finite-box size[S26] was added, using the SPC/E viscosity $\eta = 0.66$ cP.

Convergence tests

This section describes several tests to ensure our simulation protocol was statistically converged and did not suffer from finite-size effects.

Simulation box size Figure S7 shows the PMF of the large 24.82 Å cubic box comprising 6 NaCl ion pairs and 332 waters used in this work and the 12.42 Å cubic box of one ion pair and 62 waters used in validation tests.

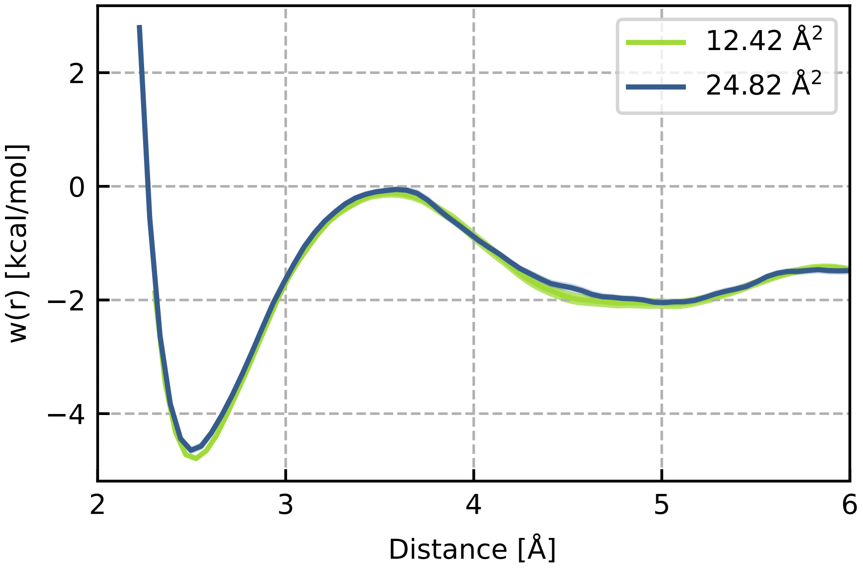

Figure S7: Comparison of finite size effects for small and large box sizes comprising a single ion pair and 1 M solution respectively.

Sampling - thermodynamic integration vs RDF To ensure that all regions along the inter-ion separation coordinate were sufficintly sampled, we compare a thermodynamic integration scheme as described in[S27] to the RDF approach used in this work. Figure S8 shows the PMF obtained from RDF and thermodynamic integration (TI) for the RPA model, showing the equivalence of the RDF approach for a 1 M system and thermodynamic integration for 1 ion pair within their own statistical errors.

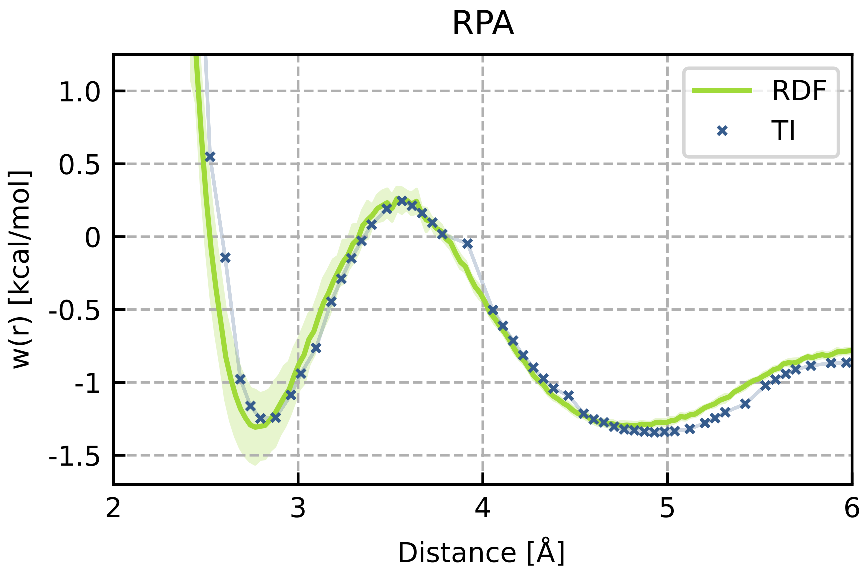

Figure S8: Comparison of thermodynamic integration and RDF approaches for the RPA model.

Simulation time

One of the major advantages of machine learning potentials is the much greater timescales accessible during simulations than for standard *ab initio* methods. Here we compute the PMF using one of the MLPs over a time period of 500 ps, for 2 replicates, a time period at the extreme upper end of that accessible for *ab initio* simulations (Note that for the hybrid and wavefunction methods even this timescale is unfeasible). In figure S9, we compare this with the ease in which the PMF can be converged over 10 replicates each of over 3 ns with the MLP.

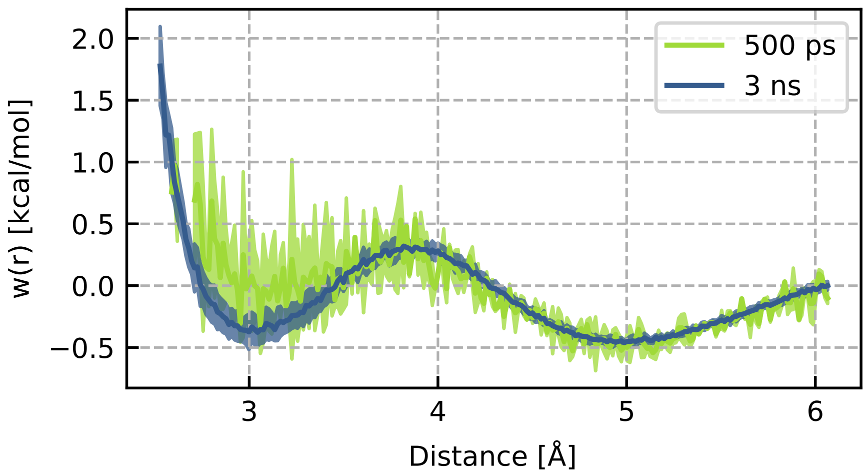

Figure S9: Comparison of PMF obtained from MLP simulations for 500 ps vs fully converged MLP simulations of 10 replicates of 3 ns each.

Additional Results

Diffusion Coefficients

The water self-diffusion coefficients were computed as a function of concentration for the MP2 and RPA models. Figure S10 shows the computed diffusion coefficients for MP2 and RPA compared with various values from literature. In all cases the self-diffusion coefficient D has been normalised with respect to the self-diffusion coefficient in bulk water with no ions (D_0).

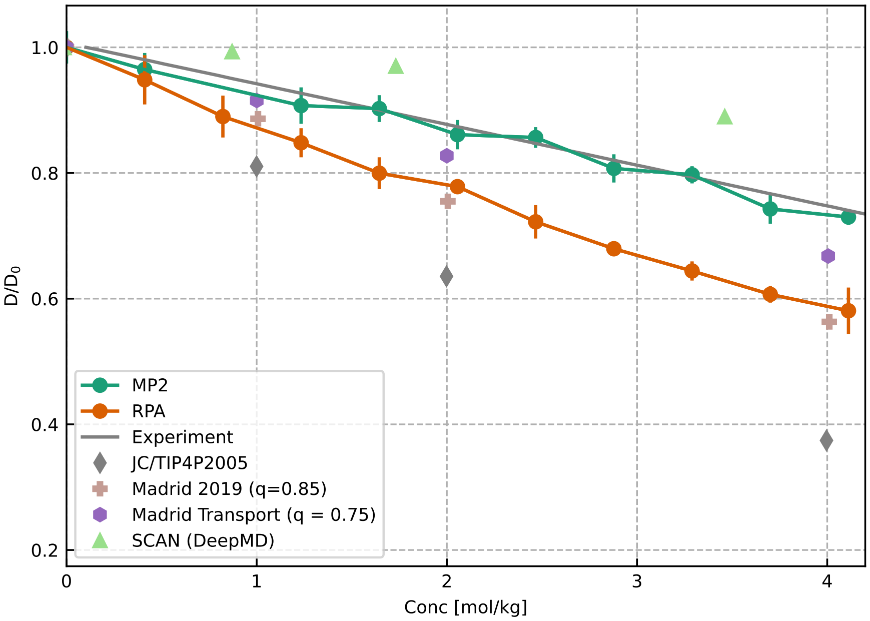

Figure S10: Normalised self-diffusion coefficient of water as a function of concentration for the MP2 and RPA models trained in this work and compared experiment (298 K) to various classical force-fields (298.15)[S28] and a DeepMD potential based on SCAN (330 K).[S29]

PMF Insights

Ion-ion binding energy: In order to explore the effect of ion-ion interactions on the PMF, we computed the binding curve of a Na Cl ion pair in vacuum using the reference electronic structure method for each model. The energy was computed for 100 ion separation distances. The binding energy was obtained from the minimum in the binding curve after alignment of the curves at 3.5 Å (inside the transition state for all models), and the curves are shown in Figure S11.

Ion-water interaction energy To obtain a proxy for the ion-water interactions we computed the energy of interaction for an ion pair in water at the 3 regions of interest – CIP, TS and SSIP. This interaction energy of the ion pair with water E_{IW} is given by

$$E_{IW} = E_{sys} - E_W - E_G \tag{S2}$$

where E_{sys} is the complete interacting system, E_W is the energy of the water with the ion-

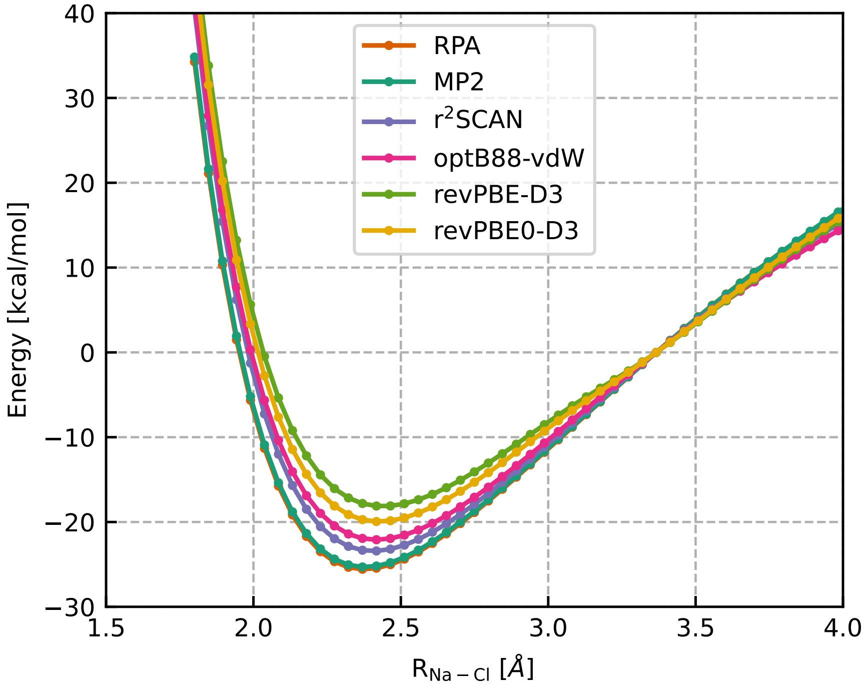

Figure S11: Na^+ Cl^- ion-ion interaction curve aligned at 3.5 Å for the various electronic structure methods used in this paper.

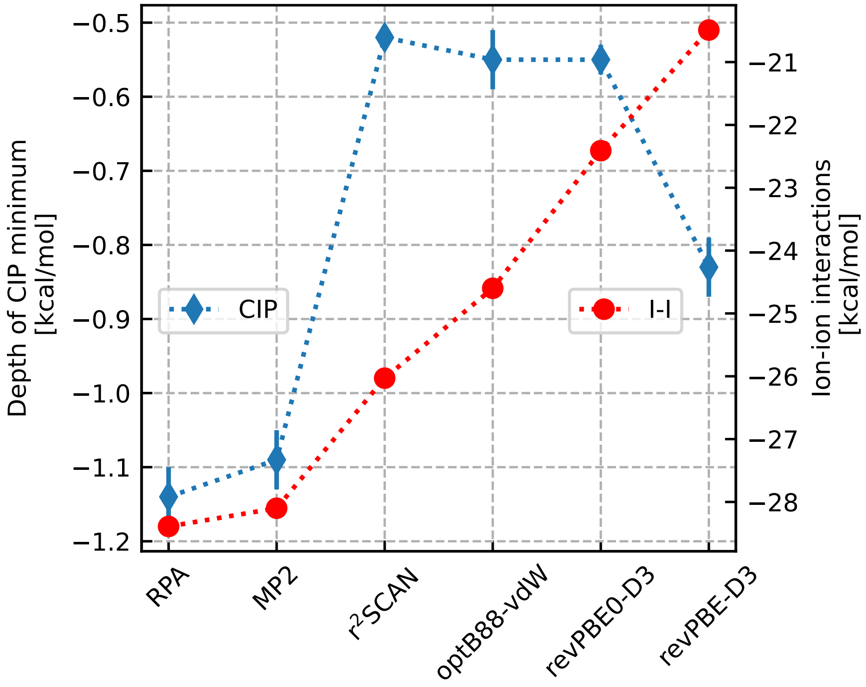

Figure S12: Relationship between CIP well depth and strength of ion-ion interactions.

pair removed and E_G is the energy of the gas-phase ion-pair. Column 3 of Table S2 shows the ion-water interaction energies for each functional. Moreover, the interaction energy of a single ion in water was also computed in a similar manner, where the interaction energy of the ion of identity X with water E_{XW} is given by:

$$E_{XW} = E_{sys} - E_W - E_G X \tag{S3}$$

where again E_{sys} is the complete interacting system, E_W is the energy of the water with the ion removed and E_G is the energy of the gas-phase ion. Table S2 shows the interaction energy of an individual Na^+/Cl^- ion with water. revPBE-D3 has the strongest Na^+ - water interaction since it is a GGA, with both delocalisation error and the D3 correction resulting in overbinding.[S30] Inclusion of Hartree-Fock exchange in revPBE0-D3 reduces this overbinding. RPA and MP2 show very similar behaviour with moderate Na^+ - water interaction but strong Cl^- - water interaction. Meanwhile most DFT functionals find it hard to agree with RPA or MP2 on the Cl^- - water interactions. This is a well known problem since it is difficult to localise the extra electron. This issue is worst for GGAs (revPBE-D3), but is improved with meta-GGA (r^2SCAN) and vdW-inclusive (optB88-vdW) functionals[S31]

Table S2: Individual ion/water (Na^+-W/Cl^--W) interaction energies and total ion-pair-water interaction energies ([Na^+ - Cl^-] - W)

Model	Na^+-W (kcal/mol)	Cl^--W (kcal/mol)	[Na^+ - Cl^-] - W (kcal/mol)
RPA	-173.78	4.79	-158.81
MP2	-172.31	3.21	-159.77
r^2SCAN	-169.29	8.83	-151.81
optB88-vdW	-170.95	5.05	-152.78
revPBE-D3	-175.65	12.68	-144.97
revPBE0-D3	-172.45	13.28	-146.72

Additional RDFs Figures S13, S14 and S15 summarise all of the RDFs computed in this work. Figure S13 shows the bulk water RDFs. Figures S15 and S14 show the ion-water and water-water RDFs respectively for the system of one NaCl ion pair in 95 waters.

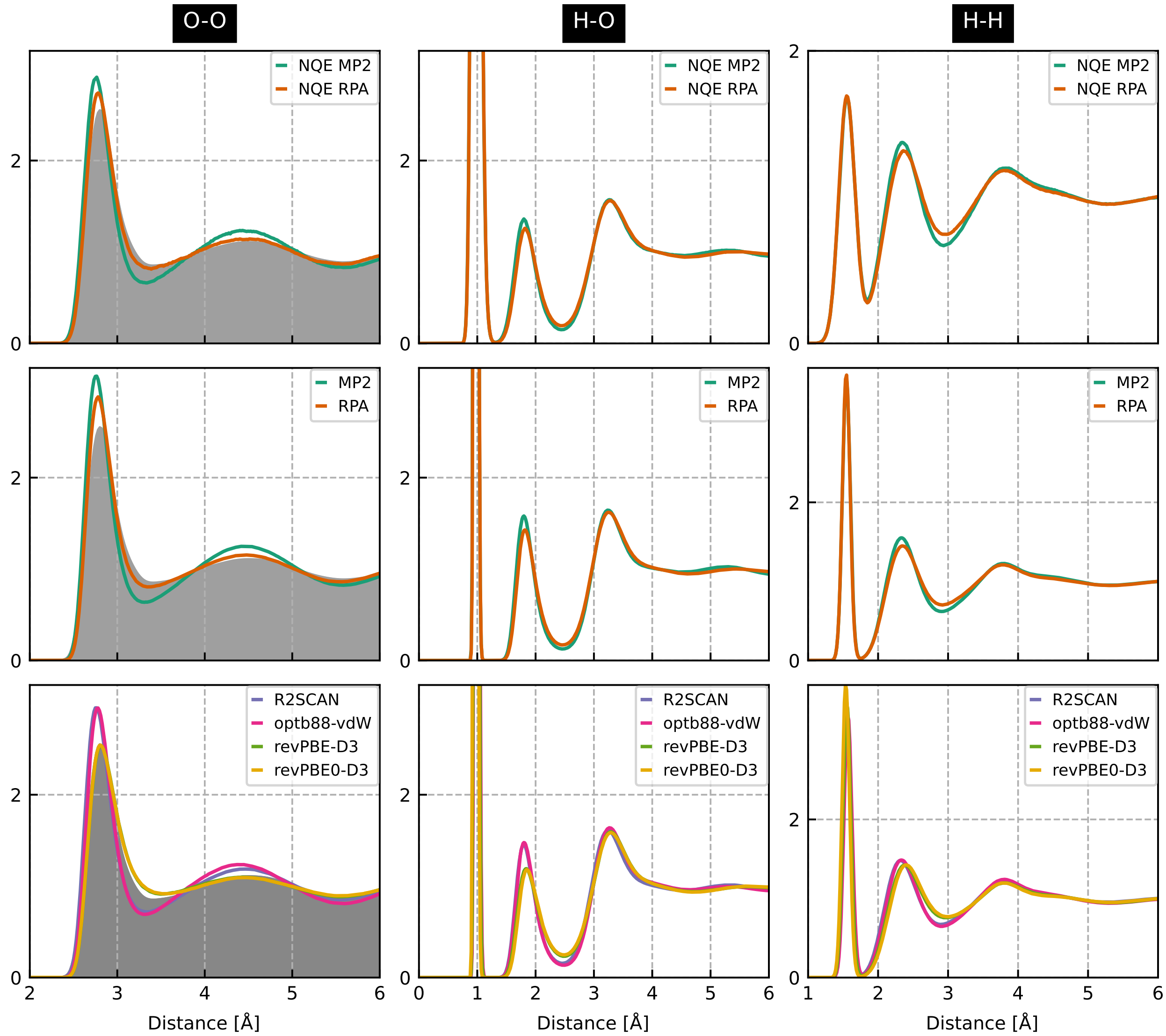

Figure S13: Water - water RDFs for bulk water.

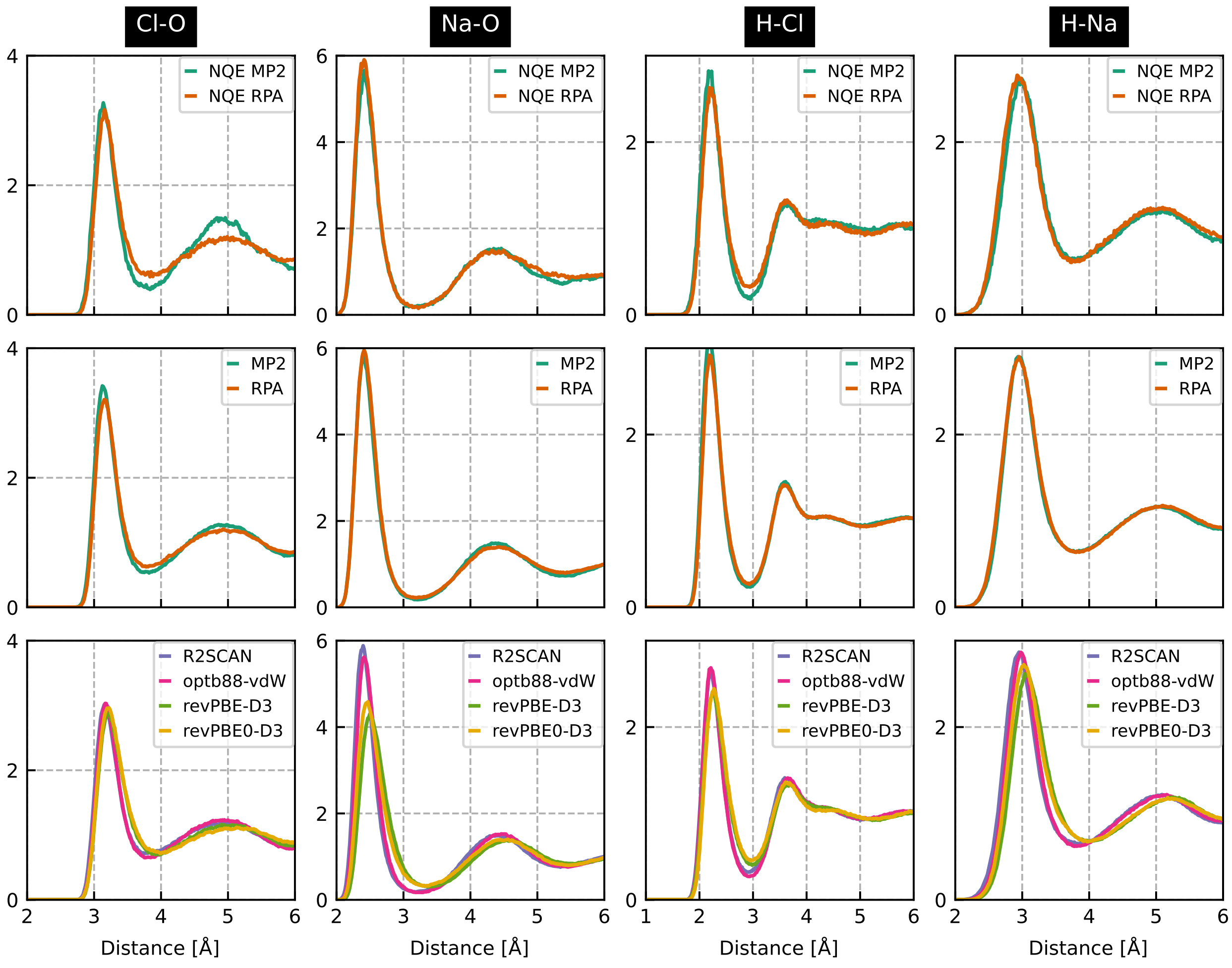

Figure S14: Ion - water RDFs for one NaCl in a 95 water box.

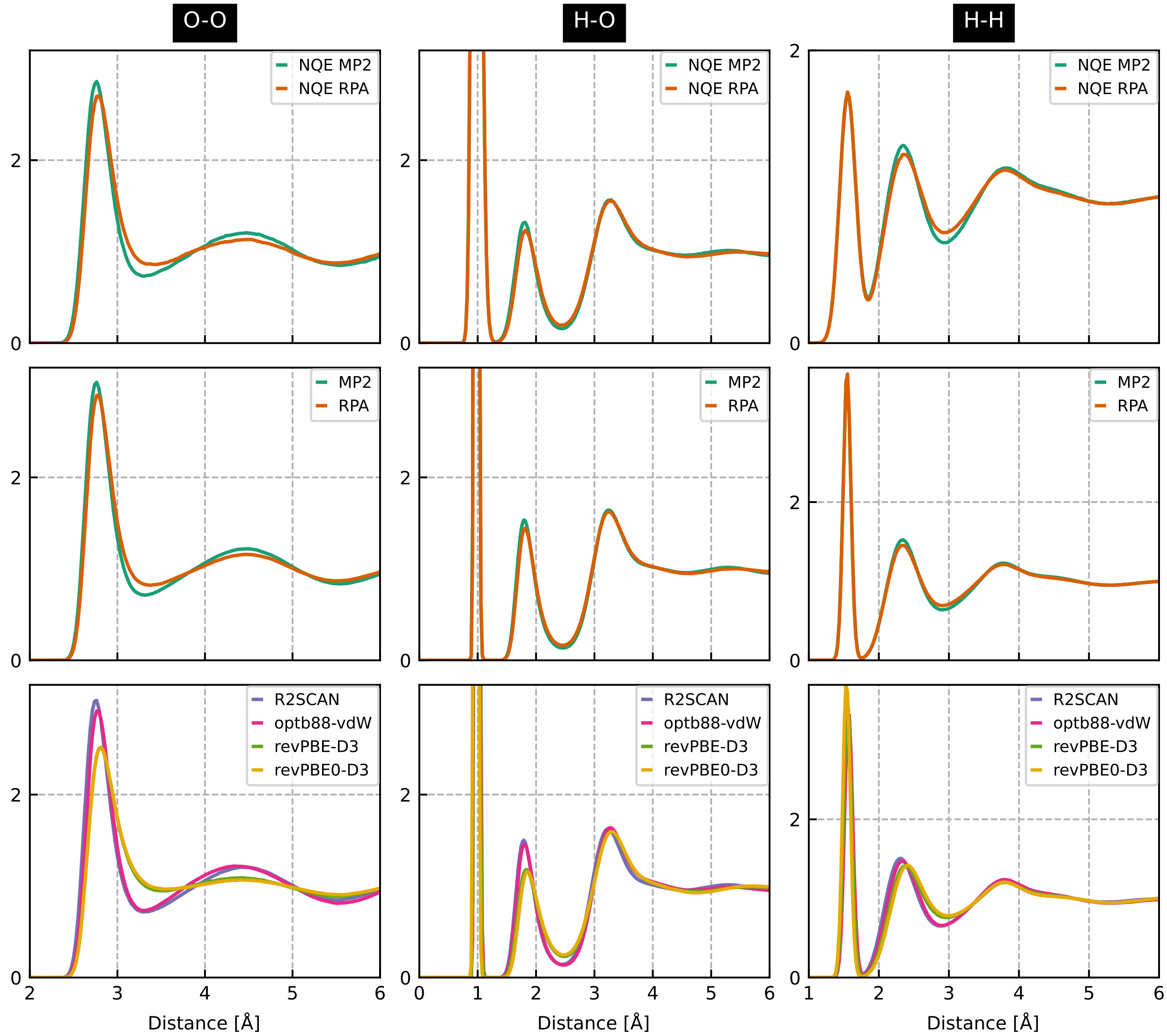

Figure S15: Water - water RDFs for one NaCl in a 95 water box.

References

(S1) Kühne, T. D.; Iannuzzi, M.; Del Ben, M.; Rybkin, V. V.; Seewald, P.; Stein, F.; Laino, T.; Khaliullin, R. Z.; Schütt, O.; Schiffmann, F. et al. CP2K: An electronic structure and molecular dynamics software package - Quickstep: Efficient and accurate electronic structure calculations. *J. Chem. Phys.* **2020**, *152*, 194103.

(S2) Goedecker, S.; Teter, M. Separable dual-space gaussian pseudopotentials. *Phys. Rev. B* **1996**, *54*, 1703–1710.

(S3) VandeVondele, J.; Hutter, J. Gaussian basis sets for accurate calculations on molecular systems in gas and condensed phases. *J. Chem. Phys.* **2007**, *127*, 114105.

(S4) Perdew, J. P.; Burke, K.; Ernzerhof, M. Generalized gradient approximation made simple. *Phys. Rev. Lett.* **1996**, *77*, 3865–3868.

(S5) Zhang, Y.; Yang, W. Comment on "generalized gradient approximation made simple". *Phys. Rev. Lett.* **1998**, *80*, 890.

(S6) Furness, J. W.; Kaplan, A. D.; Ning, J.; Perdew, J. P.; Sun, J. Accurate and numerically efficient r2SCAN meta-generalized gradient approximation. *J. Phys. Chem. Lett.* **2020**, *11*, 8208–8215.

(S7) Sun, J.; Ruzsinszky, A.; Perdew, J. Strongly Constrained and Appropriately Normed Semilocal Density Functional. *Phys. Rev. Lett.* **2015**, *115*, 036402.

(S8) Klimeš, J.; Bowler, D. R.; Michaelides, A. Chemical accuracy for the van der Waals density functional. *J. Phys. Condens. Matter* **2009**, *22*, 022201.

(S9) Adamo, C.; Barone, V. Toward reliable density functional methods without adjustable parameters: The PBE0 model. *J. Chem. Phys.* **1999**, *110*, 6158–6170.

(S10) Grimme, S.; Antony, J.; Ehrlich, S.; Krieg, H. A consistent and accurate ab initio parametrization of density functional dispersion correction (DFT-D) for the 94 elements H-Pu. *J. Chem. Phys.* **2010**, *132*, 154104.

(S11) Guidon, M.; Hutter, J.; VandeVondele, J. Auxiliary density matrix methods for Hartree-Fock exchange calculations. *J. Chem. Theory Comput.* **2010**, *6*, 2348–2364.

(S12) Lippert, G.; Hutter, J.; Parrinello, M. The Gaussian and augmented-plane-wave density functional method for ab initio molecular dynamics simulations. *Theor. Chem. Acc.* **1999**, *103*, 124–140.

(S13) Bussy, A.; Schütt, O.; Hutter, J. Sparse tensor based nuclear gradients for periodic Hartree-Fock and low-scaling correlated wave function methods in the CP2K software package: A massively parallel and GPU accelerated implementation. *J. Chem. Phys.* **2023**, *158*, 164109.

(S14) Ye, H. Z.; Berkelbach, T. C. Correlation-consistent gaussian basis sets for solids made simple. *J. Chem. Theory Comput.* **2022**, *18*, 1595–1606.

(S15) Stoychev, G. L.; Auer, A. A.; Neese, F. Automatic generation of auxiliary basis sets. *J. Chem. Theory Comput.* **2017**, *13*, 554–562.

(S16) Lehtola, S. Straightforward and accurate automatic auxiliary basis set generation for molecular calculations with atomic orbital basis sets. *J. Chem. Theory Comput.* **2021**, *17*, 6886–6900.

(S17) Del Ben, M.; Schütt, O.; Wentz, T.; Messmer, P.; Hutter, J.; VandeVondele, J. Enabling simulation at the fifth rung of DFT: Large scale RPA calculations with excellent time to solution. *Comput. Phys. Commun.* **2015**, *187*, 120–129.

(S18) Schran, C.; Thiemann, F. L.; Rowe, P.; Müller, E. A.; Marsalek, O.; Michaelides, A.

Machine learning potentials for complex aqueous systems made simple. *Proc. Natl. Acad. Sci. U. S. A.* **2021**, *118*, e2110077118.

(S19) O'Neill, N.; Schran, C.; Cox, S. J.; Michaelides, A. Crumbling Crystals: On the Dissolution Mechanism of NaCl in Water. *arXiv* **2022**, 10.48550/arXiv.2211.04345.

(S20) Chen, M. S.; Lee, J.; Ye, H. Z.; Berkelbach, T. C.; Reichman, D. R.; Markland, T. E. Data-efficient machine learning potentials from transfer learning of periodic correlated electronic structure methods: Liquid water at AFQMC, CCSD, and CCSD(T) accuracy. *J. Chem. Theory Comput.* **2023**, *19*, 4510–4519.

(S21) Pan, S. J.; Yang, Q. A survey on transfer learning. *IEEE Trans. Knowl. Data Eng.* **2010**, *22*, 1345–1359.

(S22) Behler, J. Atom-centered symmetry functions for constructing high-dimensional neural network potentials. *J. Chem. Phys.* **2011**, *134*, 74106.

(S23) Singraber, A.; Morawietz, T.; Behler, J.; Dellago, C. Parallel multistream training of high-dimensional neural network potentials. *J. Chem. Theory Comput.* **2019**, *15*, 3075–3092.

(S24) Jorgensen, W. L.; Chandrasekhar, J.; Madura, J. D.; Impey, R. W.; Klein, M. L. Comparison of simple potential functions for simulating liquid water. *J. Chem. Phys.* **1983**, *79*, 926–935.

(S25) Quaranta, V.; Hellström, M.; Behler, J. Proton-transfer mechanisms at the water-ZnO interface: The role of presolvation. *J. Phys. Chem. Lett.* **2017**, *8*, 1476–1483.

(S26) Yeh, I. C.; Hummer, G. System-size dependence of diffusion coefficients and viscosities from molecular dynamics simulations with periodic boundary conditions. *J. Phys. Chem. B* **2004**, *108*, 15873–15879.

(S27) Sprik, M.; Ciccotti, G. Free energy from constrained molecular dynamics. *J. Chem. Phys.* **1998**, *109*, 7737–7744.

(S28) Blazquez, S.; Conde, M. M.; Vega, C. Scaled charges for ions: An improvement but not the final word for modeling electrolytes in water. *J. Chem. Phys.* **2023**, *158*, 054505.

(S29) Panagiotopoulos, A. Z.; Yue, S. Dynamics of aqueous electrolyte solutions: Challenges for simulations. *J. Phys. Chem. B* **2023**, *127*, 430–437.

(S30) Bryenton, K. R.; Adeleke, A. A.; Dale, S. G.; Johnson, E. R. Delocalization error: The greatest outstanding challenge in density-functional theory. *Wiley Interdiscip. Rev. Comput. Mol. Sci.* **2023**, *13*, e1631.

(S31) DelloStritto, M.; Xu, J.; Wu, X.; Klein, M. L. Aqueous solvation of the chloride ion revisited with density functional theory: impact of correlation and exchange approximations. *Phys. Chem. Chem. Phys.* **2020**, *22*, 10666–10675.
